# Thyroid Hormone Sensitizes the Imprinting-Associated Induction of Biological Motion Preference in Domestic Chicks

**DOI:** 10.3389/fphys.2018.01740

**Published:** 2018-12-18

**Authors:** Momoko Miura, Naoya Aoki, Shinji Yamaguchi, Koichi J. Homma, Toshiya Matsushima

**Affiliations:** ^1^Department of Biology, Faculty of Science, Hokkaido University, Sapporo, Japan; ^2^Department of Life and Health Sciences, Faculty of Pharmaceutical Sciences, Teikyo University, Tokyo, Japan

**Keywords:** biological motion, imprinting, thyroid hormone, chicks, sensitive period

## Abstract

Filial imprinting is associated with induction of predisposed preference to animations that bear visual features of Johansson's biological motion (BM), and the induction is limited to a few days after hatching. As thyroid hormone (3,5,3′-triiodothyronine, T_3_) plays a critical role in determining the sensitive period of imprinting, we examined if exogenously applied T_3_ (or iopanoic acid, IOP; a selective inhibitor for converting enzymes) could also sensitize (or desensitize) the BM induction. Chicks were trained by using a non-BM stimulus (rotating red toy) according to a conventional imprinting procedure. Trained chicks were tested for preference to a point-light BM animation (walking chick) over a non-BM animation (linear motion), and for the preference for the familiarized stimulus (red toy) over an unfamiliar one (yellow toy). In 1-day chicks, those injected with IOP showed significantly lower scores than controls on both BM and imprinting tests. In 4-days chicks, those injected with T_3_ showed higher scores than control, but the difference in BM score was not significant. Imprinting and the accompanying T_3_ surge may be necessary for the predisposed BM preference to appear in 1-day chicks. Even after the conventional sensitive period is over, exogenous T_3_ can partly re-sensitize the BM preference as it does imprinting.

## Introduction

Filial imprinting constitutes the earliest step of the social attachment formation in precocial birds such as chickens and ducks (Lorenz, 1935; Hess, 1958; Matsushima et al., 2003; Horn, 2004). We have recently reported critical roles played by thyroid hormone (3,5,3′-triiodothyronine, T_3_) (Yamaguchi et al., 2012). Imprinting upregulates gene expression of the converting enzyme (type 2 iodothyronine deiodinase, Dio2) in newly-hatched (1-day old) chicks. Infusion of exogenous T_3_ (intra-venous injection) augments the imprinting score in 1-day chicks, and it reopens the sensitive period even in 4-days old chicks. We have argued that the imprinting primes the memory formation mechanism associated with imprinting. Once successfully trained, chicks can be re-imprinted to novel object for a substantially longer period than the sensitive period assumed so far. Furthermore, thyroid hormone would reorganize the neural mechanisms for visual perception. The chicks could thus develop durable social cohesion selectively to live animals such as their mother hen, even after they were initially imprinted to non-animate artifact.

Accordingly, newly-hatched chicks have an innately predisposed preference to Johansson's biological motion (BM) even without any visual experiences (Vallortigara et al., 2005). When exposed to motion pictures composed of light points, 1-day old chicks developed a distinct BM preference even when the picture was not of a BM nature (Miura and Matsushima, 2012); a video clip composed of randomly moving light points was similarly effective, but the induction of the BM preference did not occur in aged chicks (5-days old) suggesting a sensitive period as for conventional imprinting. When induced by non-specific motion pictures, the BM preference was tightly associated with imprinting (Miura and Matsushima, 2016) such that those chicks with a higher BM preference showed a higher imprinting score. Furthermore, we found a significant positive correlation between the induced BM score and the level of Dio2 expression in telencephalon of 1-day old chicks (Takemura et al., 2018). The BM preference is associated with imprinting probably via thyroid hormone actions, however, direct evidence is not yet available as to the causal relationships among these events.

In this study, we experimentally manipulated the thyroid hormone level and examined if the BM preference is accordingly controlled. The 1-day old chicks, which have an endogenously high level of T_3_ (Yamaguchi et al., 2012), were treated with systemic injection of Dio2 inhibitor (iopanoic acid, IOP). The 4-days old chicks with a low endogenous T_3_ level were supplemented with exogenous applied T_3_. These chicks and the respective control groups were trained by non-BM object (red toy) and tested for BM preference and imprinting scores.

## Materials and Methods

Fertilized eggs of white leghorn strain were purchased from a local hatchery and incubated in darkness until hatching. A total of 46 chicks (21 males and 25 females) were used. Of these, we discarded 4 chicks (3 males and 1 females) that did not walk in training and tests. We merged both sexes, as no sex difference appeared at population level. Experiments were conducted under the guidelines and approval of the Committee on Animal Experiments of Hokkaido University (approval number: #16-0080). The guidelines are based on the national regulations for animal welfare in Japan (Law for Humane Treatment and Management of Animals, after a partial amendment No. 68, 2005).

**Figure 1:**
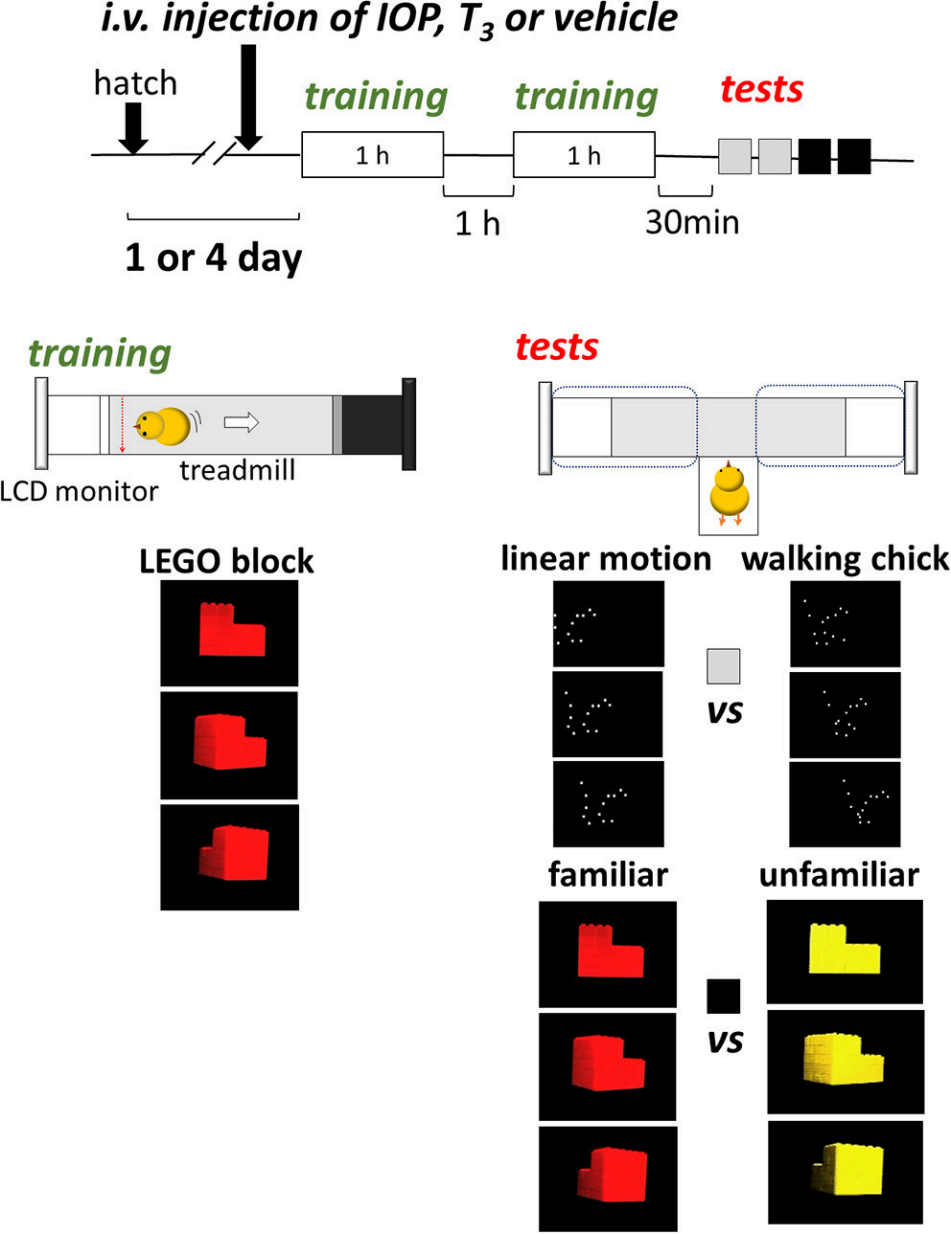
Experimental procedures. On the day of training (1- or 4-days post-hatch), chicks received intravenous injection of IOP (iopanoic acid), T_3_ (triiodo-thyronine), or respective vehicle at *ca*. 30 min before the training started. The chick was placed on a treadmill with one LCD monitor that displayed a rotating plastic toy for 1 h twice with a 1-h interval. At 30 min after the training, the chick was tested for BM preference (walking chick vs. linear chick) twice for 5 min each, and for imprinting (familiar vs. unfamiliar). The period in which the chick stayed in the left and right arm was recorded.

For training, we used an I-shaped maze with a 50-cm-long treadmill at the center and a pair of LCD monitors (size 10.4”, type LCM-T102AS, Logitec Co., Japan) at both ends (Figure 1). For display, free viewer software (APlayer, version 6.0) was used on PC. The width of the presentation was set at 9 cm on the monitor. During training, only one of the monitors was used. An infrared sensor and a transparent Plexiglass partition were placed at a point 10 cm from the active monitor. When chicks ran and hit the sensor, the treadmill moved and drew the chick backward by *ca*. 30 cm at a time. During the 1-h pause period between the two training sessions, chicks were stored in a dark incubator.

For tests, both monitors were used for binary choice. The partitions were removed and the treadmill was turned off. The subject chick was placed in a start box and allowed to freely choose between the two arms each equipped with an LCD monitor. Side of presentation alternated in two tests, and the side of the first test was counter-balanced among individuals. We recorded the total stay time in each arm (dashed lines) for a period of 5 min. The behavior of the subject chick was monitored through a CCD camera (250 kilo pixels) placed on the ceiling.

Chicks were exposed to a video clip of a toy made of red LEGO® blocks rotating along its vertical axis (see Supplementary Videos (LEGO block (red), LEGO block (yellow), Walking chick (white) and Linear motion (white)). For BM scores, we used a point-light animation that mimicked a “walking chick” used in our previous study (Miura and Matsushima, 2016; Takemura et al., 2018). One frame of the “walking chick” animation was used to generate “linear motion” of a constant speed from the left to the right of the screen. For imprinting scores, we tested chicks using the red toy video (“familiar”) and a yellow toy (“unfamiliar”). For statistical analysis, R (version 2.12.0) was used for student *t*-test for comparisons between two groups. We also constructed generalized linear models (GLMs) and evaluated these models based on AICs.

Single intravenous injection of T_3_, IOP or respective vehicle (200 μl per chick) was accomplished by using 26G hypodermic injection needle to the leg tibia vein at *ca*. 30 min before the beginning of training. IOP (Tokyo Chemical Industry) was dissolved in 0.05M NaOH solution at 1 mM, and then rebuffered to pH = 8.5 by 0.2M HCl. T_3_ (Sigma-Aldrich) was dissolved in 0.0002M NaOH and 0.9% NaCl at 10 μM.

## Results

**Figure 2:**
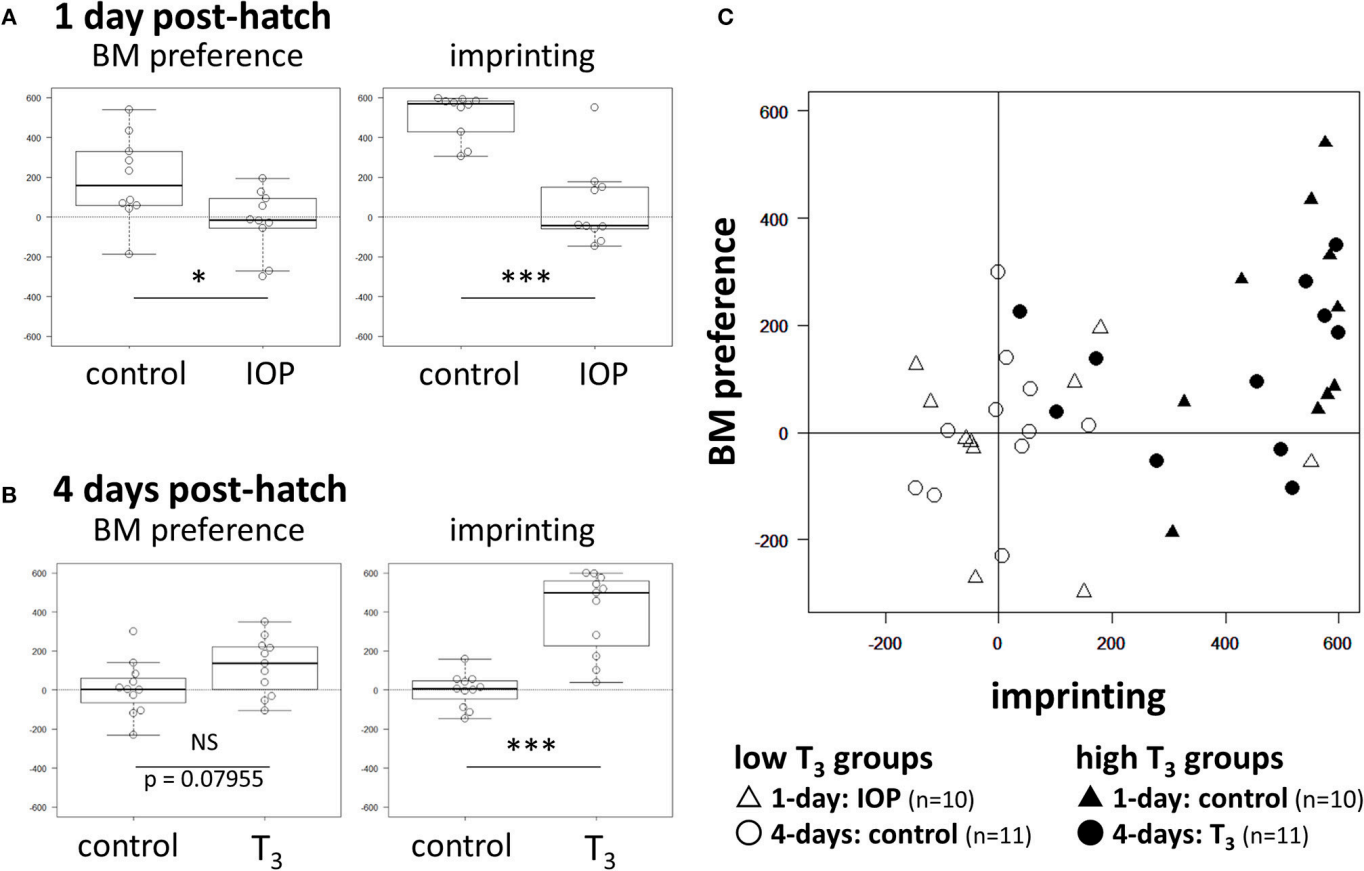
Effects of IOP and T_3_ injection on BM preference and imprinting score. **(A)** Results of 1-day chicks. **(B)** Results of 4-days chicks. NS (no significant difference), ^*^(*p* < 0.05), ^**^(*p* < 0.01), ^***^(*p* < 0.001). **(C)** BM score of individual chick was plotted against the imprinting score. Open symbols denote the low T_3_ groups; day-1 chicks with IOP injection (Δ), day-4 control chicks (◦). Filled symbols denote the high T_3_ groups; day-1 control (▴), day-4 chicks with T_3_ injection (•).

In 1-day old chicks, IOP groups showed significantly lower scores both in BM preference (*t* = −2.4784, *df* = 18, *p* = 0.023) and imprinting (*t* = −6.0682, *df* = 18, *p* < 10^−5^) than control that received vehicle injection (Figure 2A; see Supplementary Table 1 for individual data). In 4-days old chicks, the T_3_ group showed higher BM scores than control with a suggestive but not significant difference (*t* = 1.8473, *df* = 20, *p* = 0.080). On the other hand, imprinting scores were significantly higher in the T_3_ group (*t* = 5.8393, *df* = 20, *p* < 10^−4^) (Figure 2B). We conclude that T_3_ is necessary and sufficient (at least partially) for the induction of BM preference in successfully imprinted chicks.

To examine if BM preference is associated with imprinting depending on the T_3_ level, we plotted the individual BM preference score against the imprinting score after merging data obtained from these four groups (Figure 2C). In low T_3_ groups (open symbols), both imprinting scores (X-axis) and BM preference scores (Y-axis) were distributed around 0. In high T_3_ groups (filled symbols), on the other hand, those chicks with a higher imprinting score tended to show a higher BM score. Further statistical analysis using GLM (generalized linear model) revealed that interaction term of the T_3_ level and the imprinting score gave the appropriate account of the BM preference; see Supplementary Statistics (Table 2) for details. We conclude that thyroid hormone sensitizes the induction of BM preference in a manner associated with the degree of imprinting.

## Discussion

We examined the effects of experimentally manipulated T_3_ on BM preference and imprinting scores. In high T_3_ groups, those chicks with a high imprinting score tended to show a high BM preference in close accordance with our previous reports (Miura and Matsushima, 2016; Takemura et al., 2018). We may reasonably suppose that the thyroid hormone reorganized mechanisms responsible for visual perception. We must however notice that chicks with a high BM score always had a high imprinting score, but those with a high imprinting score sometimes failed to show a high BM preference (Figure 2C). In addition, the BM preference scores were generally lower in magnitude than the imprinting scores (Miura and Matsushima, 2016; Takemura et al., 2018). T_3_ may primarily act on the brain mechanisms responsible for imprinting (such as those in intermediate medial mesopallium, IMM) (Horn, 2004), which subsequently sensitizes the BM preference through some unspecified downstream mechanisms. Alternatively, our test procedure for the BM preference could be simply not optimal and we underestimated it. Further efforts must be paid to develop better procedure to measure the BM preference.

The neural substrate for BM preference has not yet been specified, but several candidate areas have been suggested. Using an immediate early gene (c-Fos) as marker, Mayer et al. (2016a,b) found septum, hypothalamus, and amygdaloid areas (arcopallium and nucleus taeniae of the amygdala; Arco and TnA) are selectively activated by visual exposure to live conspecifics. Though IMM does not have direct projections to most of these limbic areas, Arco may poly-synaptically mediate the actions of IMM down to nucleus accumbens, septum, hypothalamic nuclei, and midbrain tegmentum including optic tectum (Csillag, 1999; Montagnese et al., 2004; Hanics et al., 2017; Xin et al., 2017). This scenario fits well with a more general idea that detection of biologically important visual inputs (such as facial emotions) occurs through fast-but-robust processes in the sub-cortical (sub-pallial) pathway (Inagaki and Fujita, 2017). Alternatively, dorsal projections from IMM actions to the hyperpallium (intermediate hyperpallium apicale, IMHA; Aoki et al., 2015) may also receive visual inputs from the pallial thalamo-fugal area such as visual Wulst (Nakamori et al., 2010). Reciprocal interactions between IMHA and IMM (both show a high level of Dio2 expression; Yamaguchi et al., 2012) may be responsible for the associated BM preference and imprinting sensitized by T_3_. It is critically important to specify the brain areas responsible for the predisposed BM preference.

## Author Contributions

TM conceived the study. TM, NA, SY, KH, and TM designed the experiment. MM accomplished the experiment. MM and TM analyzed the data. TM wrote the draft of manuscript. MM, NA, SY, and KH revised the manuscript.

### Conflict of Interest Statement

The authors declare that the research was conducted in the absence of any commercial or financial relationships that could be construed as a potential conflict of interest.

## Supplementary Material

Supplementary Table 1 Behavioral data of individuals.

Supplementary Statistics Statistical analysis (GLM analysis) of the data shown in Figure 2C.

Supplementary Videos “**Video 1:** LEGO block (red)”, “**Video 2:** LEGO block (yellow), “**Video 3:** Walking chick (white)”, “**Video 4**: Linear motion (white)”: WMV type video clip data used for training and tests. Chicks were trained using “LEGO block (red)”, and the BM preference was tested in binary choice between “Walking chick (white)” and “Linear motion (white)”. Thereafter, the imprinting was tested in binary choice between “LEGO block (red)” as familiar and “LEGO block (yellow) as unfamiliar.”
