# Supplementary material for: Thyroid Hormone Sensitizes the Imprinting-Associated Induction of Biological Motion Preference in Domestic Chicks
Source: Front Physiol. 2018 Dec 18;9:1740. doi: 10.3389/fphys.2018.01740 (PMC6305540; doi:10.3389/fphys.2018.01740)
Supplement: Supplementary Statistics — Statistical analysis (GLM analysis) of the data shown in Figure 2C. [file Table_2.DOCX]

Supplementary note on the generalized linear model (GLM) analysis on the merged 4 groups of behavioral data shown in Figure 2C.

To examine if BM preference is associated with imprinting depending on the T3 level, we plotted the individual BM preference score against the imprinting score after merging data obtained from these 4 groups (Figure 2C). We assumed full and null GLM (generalized linear models) as follow. These models had AIC (Akaike Information Criteria) ~ 559.8 (full model) and 552.9 (null model) respectively.

- Full model:

(*BM score*) = α_0_ + α_1_*(*imprinting score*) + α_2_*(*T3*) + α_3_*(*imprinting score* x *T3*) [AIC = 559.8]

- Null model:

(BM score) = α_0_ [AIC = 552.9]

Here, the variables in the formula are defined as:

- (*imprinting score*) represents the integer values shown as “imprinting” in Figure 2.
- (*T3*) is LOW for 1-day:IOP and 4-days control, or it is HIGH for 1-day: control and 4-days:T3.
- (*imprinting score* x *T3*) denotes the interaction between the two variables.

Among the all possible combinations, the following two models showed the lowest AIC values. The other models had higher AIC values and not considered here.

1. (*BM score*) = α_0_ + α_3_*(*imprinting score* x *T3*) [AIC=550.9]

Coefficient α_3_ was significant and the probability that α_3_ ≤ 0 was lower than *p* = 0.05.

1. (*BM score*) = α_0_ + α_1_*(*imprinting score*) + α_3_*(*imprinting score* x *T3*) [AIC = 550.9] Similarly, α_1_ was significant. On the other hand, α_3_ was not significant, and the probability that α_3_ ≤ 0 was higher than *p* = 0.05.

Considering that (*imprinting score*) implicitly includes (*T3*), we conclude that thyroid hormone sensitizes the induction of BM preference in a manner associated with the degree of imprinting.
